# Feasibility, acceptability and preliminary effectiveness of a mental health drop-in centre for the siblings of young people attending a paediatric hospital

**DOI:** 10.1177/13674935231206895

**Published:** 2023-10-18

**Authors:** Sophie D Bennett, Natalia Rojas, Matteo Catanzano, Anna Roach, Brian CF Ching, Anna E Coughtrey, Isobel Heyman, Holan Liang, Lucy Project Team, Roz Shafran

**Affiliations:** 1 UCL Great Ormond Street Institute of Child Health, 4919 University College London, London, UK; 2 Psychological and Mental Health Services, 4956 Great Ormond Street Hospital for Children NHS Foundation Trust, London, UK

**Keywords:** Siblings, mental health, chronic disease, pediatrics, quality of life

## Abstract

Siblings of children with long-term conditions (LTCs) can have significantly elevated mental health needs, but these are often overlooked. A pragmatic single-arm feasibility pilot assessed feasibility, acceptability and preliminary effectiveness of a drop-in centre in a paediatric hospital addressing mental health needs of patients with LTCs, their carers and siblings. The drop-in centre accepted self-referral and supplemented existing provision offering a suite of interventions, including signposting, diagnostic assessments and/or guided self-help. This paper reports on feasibility, acceptability and preliminary outcomes of this centre for siblings. Eighteen siblings aged 2–17 used the centre. Sixteen of their parents completed the Strengths and Difficulties Questionnaires at baseline and 6 months post-baseline, and ten completed parent-reported PedsQL across two time points. Preliminary effectiveness results demonstrated a decrease in mental health symptoms with large effect size (score reduction of 3.44, 95% CI [1.25, 5.63], d = 0.84) and small effect on quality of life, with scores increasing from a median of 69.91, 95% CI [53.57, 91.67], to a median of 80.44, 95% CI [67.39, 89.13], r = 0.11 for these siblings. 88% of parents were satisfied with this provision for their sibling child. This study highlights the feasibility and value of assessing siblings for emotional and behavioural difficulties and providing them with an accessible, effective and acceptable intervention.

## Introduction

Long term conditions (LTC) are defined as diagnosed physical health conditions lasting for a minimum of 3 months for which a cure is unlikely and result in limitations in ordinary activities and increased use of health services (Moore et al., 2019). Siblings of children with an LTC can experience a number of challenges, such as reduced time spent with parents (Wilkins and Woodgate, 2005), social challenges (Bluebond-Langner, 2000), family tension (Kvist et al., 2013; Mailick Seltzer et al., 2001) and observation of sibling medical emergencies (Knecht et al., 2015). As a result, they appear to have elevated emotional and behavioural difficulties (Kelada et al., 2021; Vermaes et al., 2012). The purpose of the present study was to evaluate the feasibility, acceptability and preliminary outcomes of a drop-in centre in a paediatric hospital. The drop-in centre was designed to improve access to effective treatments for mental health difficulties in siblings of children with an LTC.

Psychological interventions for siblings of children with LTCs have been developed, with mixed success (McKenzie Smith et al., 2018; Mitchell et al., 2021). A meta-analysis of well-being interventions for siblings indicated that treatments significantly improved emotional outcomes and knowledge of their sibling’s health conditions but had no impact on behavioural outcomes compared to control groups (McKenzie Smith et al., 2018). Of seventeen studies, eight were considered to be of weak quality, highlighting a need for more methodologically robust studies. More recently, a meta-analysis of six parenting interventions to support siblings of children with an LTC found that such interventions may improve siblings’ emotional and behavioural adjustment (Mitchell et al., 2021).

Despite studies demonstrating interventions for siblings of children with LTCs are effective, many siblings do not have their mental health needs recognised and many do not access evidence-based interventions (Hartling et al., 2014). Brief and low-intensity interventions are typically shorter than standard therapy. Brief interventions are typically 50% or less of standard therapies; low-intensity interventions use self-help materials and can be delivered by trained practitioners or supporters rather than fully qualified cognitive behavioural therapy (CBT) therapists (Shafran et al., 2021). Such interventions can increase access to evidence-based interventions and are effective for children and young people (Bennett et al., 2019), including those with LTCs (Catanzano et al., 2020). This approach to care can be part of a ‘stepped care pathway’, in which a patient starts with a lower-intensity evidence-based treatment, with systematic monitoring of progress, and may then ‘step up’ to a higher-intensity treatment if they do not adequately respond (Kendrick and Pilling, 2012).

One effective model of increasing access to brief psychological support to young people is through drop-in centres (Barwick et al., 2013; Cheung et al., 2017; Nath et al., 2016). However, no previous research has explored their potential use in paediatric hospital settings. Co-localisation of resources may facilitate efficient service integration and joint work amongst professionals, as well as flexibility and ease of access for families (Baxter et al., 2018).

A pragmatic single-arm feasibility pilot was conducted to assess feasibility, acceptability and the preliminary impact of a drop-in centre in a paediatric hospital to address mental health needs of patients with LTCs, their carers and siblings. The drop-in centre accepted self-referral and supplemented existing provision by offering a suite of interventions, including single sessions, signposting or referral to appropriate services, a diagnostic assessment and/or guided self-help. Previous papers have reported on the feasibility and positive impact of this centre on children with LTCs and their parents (Catanzano et al., 2021; Bennett et al., 2021). No previous work has examined feasibility, acceptability and preliminary effectiveness of drop-in centres for siblings of children with LTCs within paediatric hospitals or more broadly.

## Aim

The aim is to explore feasibility, acceptability and preliminary effectiveness of a drop-in centre for supporting the mental health of siblings of children with LTCs.

## Methods

### Design

This study was conducted as part of a wider uncontrolled pilot evaluating a mental health drop-in centre situated in a national paediatric hospital offering mental health support for patients, their siblings and parents/carers (Catanzano et al., 2021). Results for patients, parents and carers are reported elsewhere (Bennett et al., 2021; Catanzano et al., 2021).

### Ethics

Ethical approval was granted by the London Riverside Research Ethics Committee (REC reference number: 16/LO/1915). Approval covered the full study, including evaluation for patients, parents and siblings.

### Population

The wider study included patients attending a national paediatric hospital in England for a long-term condition and their parents/carers and siblings. Only outcomes for siblings are reported presently. The hospital annual report from the study period states they provided more than 260,000 appointments and admissions; however, specific numbers of children with LTCs and their families are not available.

### Inclusion and exclusion criteria

Siblings had to have a common mental health need (e.g. anxiety, depression behavioural difficulties and sleep difficulties) that was interfering with current functioning. Participants (siblings and their parents) had to have a sufficient grasp of English to facilitate engagement with assessment and treatment.

Families were excluded if they were currently under the hospital’s paediatric psychology services’ care.

### Measures

Only carer-reported child mental health measures were analysed as most siblings presenting were chronologically or developmentally younger than 11 years. The primary measures needed to be suitable for the whole population, that is, covered the full range of age and ability levels. For pragmatic reasons, it was not possible to collect a wide range of secondary outcome measures suitable for different groups.

#### Mental health and quality of life

Siblings’ mental health was assessed using the Strengths and Difficulties Questionnaire (SDQ) (Goodman, 1997): a 25-item parent-reported measure for children aged 4–17 (Goodman and Goodman, 2011) with moderate test–retest reliability and good concurrent and discriminant validity (Goodman, 2001). Quality of life was assessed using the parent-report Pediatric Quality of Life Inventory (PedsQL) (Varni et al., 1999): a 23-item measure with excellent validity and reliability (Green et al., 2005). This was introduced 1 month after the start of the study to capture change in participants’ physical functioning. The appropriate form was used depending on the child’s age.

#### Acceptability

A slightly modified version of a widely used measure of service satisfaction, the CSQ-8, was completed by parents, where additional items were added (i.e. items eliciting potential improvements), some were reworded and open text was provided for some items. Responses were on a five-point scale: ‘not at all’, ‘only a little’, ‘somewhat’, ‘quite a bit’ and ‘totally’, ranked 0 to 4 (Attkisson and Greenfield, 1994).

### Procedure

One volunteer/member of staff was present at the drop-in centre with a clinical psychologist and/or psychiatrist on call at all times. Participants were flexibly recruited:

(i) A family/sibling could approach a drop-in centre staff member (‘physical drop-in’).
(ii) A family/sibling could contact drop-in centre staff by e-mail/telephone.
(iii) A staff member could approach a family/sibling in other hospital areas with a project leaflet (‘active recruitment’).
(iv) Hospital clinicians could signpost siblings/families.
(v) Hospital clinicians could refer siblings/families.

Referring clinicians and the drop-in study team informed families that the drop-in centre was a research project. Families were invited to participate and provided informed consent. Consenting families completed baseline measures (parent-reported SDQ and PedsQL) over telephone/face-to-face. Those who did not want to consent were signposted back to their physical health teams to discuss options for support where appropriate. Following this, a triage assessment was carried out in accordance with a standardised protocol over telephone/face-to-face. All siblings were then discussed in a weekly meeting with a consultant child and adolescent psychiatrist and allocated to an intervention. Intervention allocation was based on clinical judgement that considered factors including clinical risk, relationship to physical health condition, participant preference, neurodevelopmental factors, family factors and symptom severity. At no point in the study was there a waiting list for an initial triage assessment. Participants waited a maximum of 7 days to be allocated to an intervention. All measures were completed at baseline after consent and 6 months post-baseline. Outcome measures (SDQ, PedsQL and CSQ-8) were collected face-to-face/over telephone/e-mail by a researcher that was independent from intervention delivery.

### Intervention

The following interventions were available for siblings:

(i) Provision of/direction to self-help materials and/or online resources (e.g. evidence-based self-help books or websites relevant to their child’s condition like the National Autistic Society).
(ii) A neurodevelopmental assessment and/or computerised mental health diagnostic assessment: the Development and Wellbeing Assessment (DAWBA) (Goodman et al., 2000); for participants where an undiagnosed neurodevelopmental condition (e.g. autism spectrum disorder or attention-deficit hyperactivity disorder) was suspected during initial assessment.
(iii) Signposting/referral to appropriate internal/external services. Internal services included hospital Psychological Services and Family Therapy, and external services included other local child mental health services and charities.
(iv) A brief modular psychological intervention defined as up to six sessions (6 hours total) of telephone/face-to-face guided self-help based on the Modular Approach to Therapy for Children with Anxiety, Depression, Trauma, or Conduct Problems (MATCH-ADTC) (Chorpita and Weisz, 2009); provided by newly qualified clinical psychologists, a junior doctor and/or trained psychological well-being practitioners (CWPs). CWPs are individuals trained specifically in low-intensity therapies, usually through a specific program, associated with the Improving Access to Psychological Therapies initiative. Weekly group supervision was provided by a qualified clinical psychologist. The intervention was delivered to parents and/or children depending on presenting difficulty, age and intellectual ability. Further intervention details have been presented previously (Catanzano et al., 2021).

### Analysis

#### Participant characteristics and flow

Sibling participants flow through the period of the study (17 months, January 2019–June 2020) was investigated. Data collected included the following:

• numbers of siblings dropping in/referred by clinicians/parents;
• numbers of siblings consenting; numbers of siblings provided with an intervention for their own mental well-being;
• number of parents completing measures regarding siblings’ mental health and quality of life.

#### Sibling mental health and quality of life

Siblings’ median scores on mental health and quality of life measures were calculated.

#### Interventions

Category of intervention provided was assessed (provision of self-help materials, further neurodevelopmental or mental health assessment, signposting or referral to internal or external services or brief psychological therapy).

#### Satisfaction

Median CSQ-8 score was calculated, and open texts provided to open-ended questions (‘What did you like the most about the service you received from the centre?’ ‘If the information and support you received made a difference to you/your child's mental or physical health, please give details here’ and ‘What was most helpful about the information and support you received’) were summarised.

#### Preliminary effectiveness

SPSS (version 25, IBM) was used to compare baseline measurements of sibling mental health and quality of life (SDQ and PedsQL) to those collected at 6 months post-baseline. Difference scores were tested using paired samples t-tests or Wilcoxon Signed-Rank Tests depending on normality, which was assessed through Shapiro–Wilk tests and visual inspection of Histograms, Q-Q Plots and Box Plots. Effect sizes, with 95% Confidence Intervals, were calculated, these were either Cohen’s d (for paired samples t-tests), where 0.20 is considered a small effect, 0.50 a medium effect and 0.80 a large effect (Cohen, 2013), or *r* (for Wilcoxon Signed-Rank Tests), where 0.10, 0.30 and 0.5 constitute small, medium and large effects, respectively.

## Results

### Participant Flow and completion of measures

**Figure 1.**
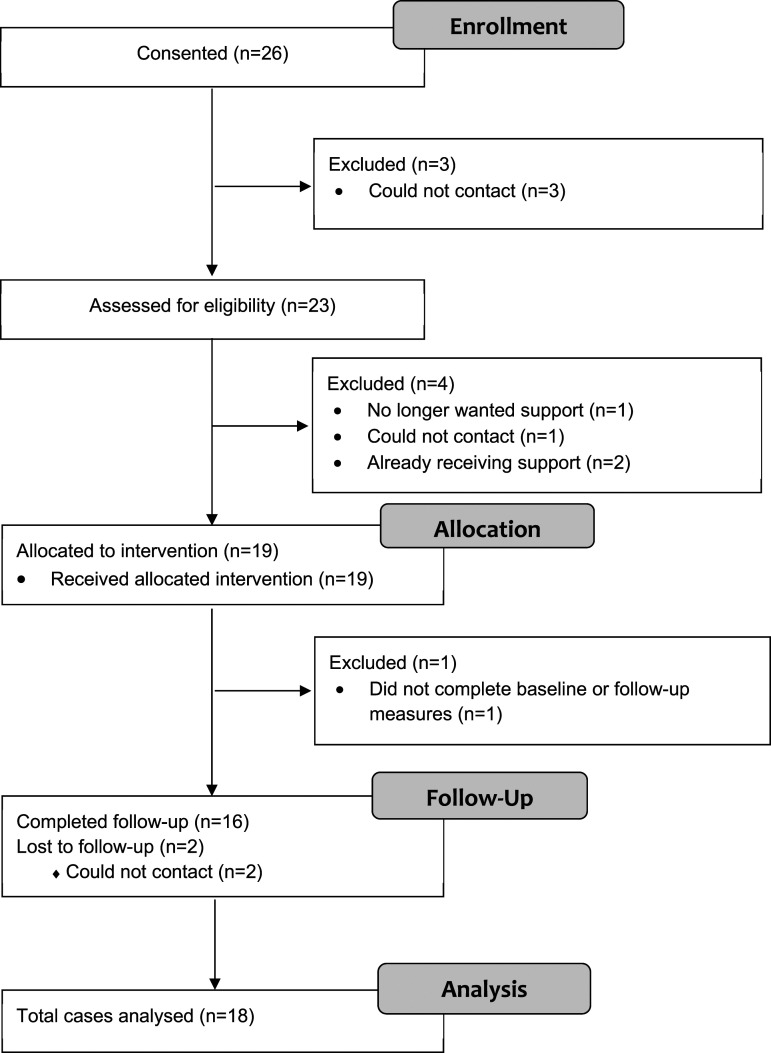
Adapted CONSORT diagram showing patient flow (siblings).

Figure 1 illustrates participants’ participation flow, with reasons for exclusion and attrition. A total of 26 siblings were consented; 16 siblings (62%) were actively recruited, 4 (15%) were recruited by email/call drop-in, 5 (19%) by physical drop-in and 1 (4%) by signposting. Twenty-three were assessed for eligibility and three could not be contacted. Of 23 assessed for eligibility, 4 were excluded as they no longer wanted support (*n* = 1), could not be contacted (*n* = 1) or were already receiving support (*n* = 2). There were 19 siblings allocated to an intervention and 18 completed baseline measures. Of 18 siblings allocated to an intervention who completed baseline measures, 89% of parents (16/18) completed a parent-reported SDQ for the siblings and 56% (10/18) a PedsQL across the two time points. SDQ response rates were higher as the PedsQL was introduced 1 month after the study had started. This meant six parents were not asked to complete it.

### Participant characteristics

Demographics of included participants (*n* = 18) are shown in Table 1. Ten (56%) participants were the only child in the family provided intervention by the centre; six (33%) had a sibling with LTCs who was also provided intervention; one (6%) had a parent who was supported for their own mental health problems through the study; and one (6%) had both a sibling with LTCs and parent who were provided intervention.

**Table 1.** Core participant demographics of the siblings of children and young people with long-term physical conditions attending the drop-in centre along with the mean and SD, median and IQR, number (*n*) and percent (%) of cases (where relevant) for all data. IMD decile = index of multiple deprivation decile.

		All siblings (*N* = 18)
Age of siblings, mean (SD)		8 (4)
IMD decile, median (IQR)		5 (6)
Sex, % (*n*)	Female	56 (10)
	Male	44 (8)
Ethnicity, % (*n*)	White	67 (12)
	Asian	11 (2)
	Black	6 (1)
	Any other mixed background	17 (3)
Primary intervention allocated to, % (*n*)*	MATCH	44 (8)
	Referral	33 (6)
	Signposting	22 (4)
	Liaison	6 (1)
Known neurodevelopmental disorder, % (*n*)	Present	22 (4)
Primary presenting complaint, % (*n*)	Anxiety	22 (4)
	Low mood	11 (2)
	Challenging behaviour	50 (9)
	Adjustment disorder	17 (3)
Index child's primary medical specialty, % (*n*)	Haematology	11 (2)
	Dermatology	6 (1)
	Rheumatology	17 (3)
	Cardiology	6 (1)
	Ears, nose and throat	28 (5)
	Urology	11 (2)
	Respiratory	6 (1)
	Neurosurgery	6 (1)
	Endocrinology	6 (1)
	Ophthalmology	6 (1)
	None specified	6 (1)

*N = 19 as one participant was both referred and signposted.

The majority of siblings attending the drop-in centre were White (12/18, 67%), primary-school aged (M = 7.94, SD = 4.07), females (10/18, 56%) and had a median Index of Multiple Deprivation (IMD) decile of five (IQR: 6), where one is most deprived and ten is least deprived. IMD is the official measure of relative deprivation for small areas in England, created through combining information from seven domains of deprivation, such as income, employment, education, health, crime, housing and environment (Department for Communities and Local Government, 2016) .

Four siblings (22%) presented with known neurodevelopmental disorders. Families reported a mean total number of three children in the household (IQR: 1).

### Mental health and quality of life

Median scores for siblings on parent-reported SDQ and PedsQL are presented in Table 2. Overall, results indicate high levels of mental health needs in our sample, with 11 of 18 (61%) scoring above clinical threshold on SDQ Total score (17+) at baseline. Specifically, 10 (56%), 9 (50%), 5 (28%) and 8 (44%) of siblings scored above the clinical threshold on SDQ Emotional, Conduct, Hyperactivity and Peer subscales, respectively. Four of eleven (36%) scored above clinical threshold for PedsQL Total score.

**Table 2.** Medians and interquartile ranges^a^ (IQR) of the Strengths and Difficulties Questionnaire (SDQ) and Pediatric Quality of Life Inventory (PedsQL) scores of the siblings at pre-intervention and 6 months post-baseline.

Measure	Pre		Post	
	*n*	Median (IQR)	*n*	Median (IQR)
SDQ Total score	18	19 (15)	16	12 (9)
Impact	17	3 (4)	15	1 (4)
Emotional	18	5 (4)	16	4 (5)
Conduct	18	3.5 (4)	16	2 (3)
Hyperactivity	18	6 (3)	16	4.5 (4)
Peer	18	3 (5)	16	1 (3)
Prosocial	18	7 (5)	15	8 (5)
PedsQL Total score	11	69.57 (37)	11	81.52 (20)
Physical functioning	9	90.63 (39)	11	93.75 (19)
Psychosocial functioning	10	57.22 (33)	11	80 (30)
Emotional functioning	11	35 (40)	11	65 (30)
Social functioning	11	80 (50)	11	90 (30)
School functioning	10	85 (35)	11	75 (23)

^a^Median and IQR are reported due to outliers, significant Shapiro–Wilk normality tests and the small sample size.

### Interventions provided

Table 1 provides a breakdown of assigned interventions for each sibling. Of note, multiple interventions were possible and so some may be represented more than once. Three siblings were referred to internal psychological services, all were accepted, with one starting treatment (seen for one session by a clinical psychologist), one declining as they no longer needed support and one offered support at a later time. Three were signposted to self-help resources, one was referred to internal hospital paediatric psychology services and another internally to Family Therapy. One sibling was both referred to their local community well-being service (Tier 2 children and young people’s mental health service) and signposted to self-help resources, with another having been recently referred to internal psychological services and so liaison work was carried out between the study team and internal psychological services. Eight siblings received adapted modular treatment, based on MATCH-ADTC (Chorpita and Weisz, 2009).

Of those eight receiving modular treatment, a median of six (IQR: 1) treatment sessions were delivered. Modules delivered to these eight were ‘Anxiety’ (1, 12.5%), ‘Depression’ (1, 12.5%) and ‘Conduct’ (6, 75%). In three (38%) participants, more than one module was used (e.g. if their primary presenting problem was low mood but anxiety symptoms interfered with treatment, a session might be spent working on such symptoms before returning to the Depression module). Within these siblings, other family members were also offered intervention. Two participants’ siblings who were hospital patients (index child) also received MATCH-ADTC, another index child was referred to internal psychological services and one participants’ parent was supported to make a referral to adult mental health service and received self-help materials.

### Acceptability – Client satisfaction questionnaire (CSQ-8)

Client Satisfaction Questionnaires were completed by parents of 16 siblings. 88% (*n* = 14) of parents were either ‘Totally’ or ‘Quite a bit’ satisfied with the provision for their sibling child. Most questions scored a median of 4 (‘Totally satisfied’) and parents were ‘grateful that we had access and could use the service’. The perceived usefulness of information/support received demonstrated a median score of ‘3’ (‘Quite a bit’) with parents highlighting the value of ‘speak[ing] to someone and go[ing] over my experiences’, ‘person to person contact’ and ‘knowing where to go next’. Parents’ reports of whether information/support made any difference to either their own or their child’s physical health demonstrated a median score of ‘1’ (‘Only a little’) but associated open text suggested positive impacts on sleep and low mood, ‘[sibling] is sleeping better and she is less tearful. She was more upset before…’; anxiety ‘she is now less anxious and worried’ and general outlook on situations ‘[they are] … more open, [sibling] was quite a closed book and he’s trying to explain it more, and not thinking about everything too much’.

### Preliminary effectiveness on mental health and quality of life outcomes

For siblings with paired SDQ scores, scores improved for all subscales between baseline and 6 months post-baseline (see Table 3). There was a mean decrease of 3.44, 95% CI [1.25, 5.63], d = 0.84 (large effect size) on SDQ total score. Impact scores also demonstrated improvement, decreasing from a median of 2, 95% CI [0,3], at baseline to a median score of 0.5, 95% CI [0,3], 6 months post-baseline, Z(14) = 1.31, r = 0.35, reflecting a medium effect size.

**Table 3.** Comparison of SDQ and PedsQL scores at baseline and 6 months follow-up.

Measure	*n*	Pre	Post	Mean difference (95% CI)	*t*/Z	Cohen’s d/*r*
		Mean (SD)/median (IQR)^a^	Mean (SD)/median (IQR)^a^			
SDQ Total score	16	18.00 (8.10)	14.56 (7.39)	3.44 (1.25, 5.63)	3.342	0.84
Impact^#^	14	2.00 (3)	0.50 (3)		1.314	0.35
Emotional	16	5.19 (2.32)	4.56 (2.71)	0.63 (−0.10, 1.35)	1.838	0.46
Conduct	16	3.81 (2.99)	3.13 (2.63)	0.69 (−0.13, 1.51)	1.789	0.45
Hyperactivity	16	6.13 (2.78)	5.13 (2.58)	1.00 (−0.17, 2.17)	1.826	0.46
Peer^#^	16	2.50 (5)	1.00 (3)		2.431	0.61
Prosocial^#^	15	6.00 (5)	8.00 (5)		1.412	0.36
PedsQL Total score^#^	10	69.91 (36.88)	80.44 (20.50)		0.357	0.11
Physical health^#^	8	84.38 (44.53)	89.07 (19.53)		0.524	0.19
Psychosocial health^#^	9	57.22 (35.83)	75.00 (30.56)		0.889	0.30
Emotional functioning^#^	10	42.50 (37.50)	65.00 (20.00)		2.409	0.76
Social functioning^#^	10	80.00 (51.25)	80.00 (35.00)		0.338	0.11
School functioning^#^	9	90.00 (38.34)	75.00 (26.66)		0.000	0

^a^Means, Standard Deviations (SDs), 95% Confidence Intervals (CIs) around the mean difference for paired t-tests and Medians, Interquartile Ranges (IQRs) for Wilcoxon Signed-Rank Tests and effect sizes (d/*r*) are shown for all participants included in the analysis.

^#^ = Wilcoxon Signed-Rank Test.

PedsQL, Pediatric Quality of Life Inventory; SDQ, Strengths and Difficulties Questionnaire.

For siblings with paired PedsQL scores, total score and all but two of the subscales demonstrated improvement from baseline to 6 months post-baseline, with one subscale remaining stable across the follow-up period and one worsening (Table 3). There was a median increase in Total PedsQL scores, reflecting better quality of life, with scores increasing from 69.91, 95% CI [53.57.91.67], to 80.44, 95% CI [67.39.89.13], with a small effect size (r = 0.11). The School Functioning subscale showed a median decrease in quality of life, with scores falling from 90.00, 95% CI [55.00, 91.67], at baseline to 75.00, 95% CI [65.00, 90.00], 6 months post-baseline, with a very small effect size (r < 0.01).

Numbers of siblings completing different types of interventions were too small to analyse these as separate sub-groups.

## Discussion

This study aimed to explore feasibility, acceptability and preliminary effectiveness of a drop-in centre for supporting the mental health of siblings of children with LTCs. The study specifically explored the number and characteristics of attending siblings, number of parents completing measures, mental health and quality of life of attending siblings, types of interventions sought and levels of satisfaction with this integrated provision. The study also sought to investigate the preliminary effectiveness of the drop-in centre intervention. Overall, a variety of interventions were sought and there was preliminary evidence that such interventions were feasible, acceptable and effective, at least in the short term.

Regarding feasibility of recruitment, actively approaching families and informing them of support for siblings may have meant that siblings were more likely to have their needs met. A meta-synthesis demonstrated that parents may not recognise or may deprioritise their own emotional needs in favour of balancing their family’s needs (Deavin et al., 2018). However, relatively small numbers of siblings attended, which may suggest that other methods of identifying need are also required, particularly as siblings may conceal their own difficulties and may not share them with parents or may feel conflicted about reporting them in front of their parents (Haukeland et al., 2015).

Only one parent did not complete baseline measures, and 16 of 18 completed follow-up, suggesting that these parent-reported measures were feasible and acceptable. However, previous research suggests that parents may underestimate emotional responses and needs of healthy siblings (Deavin et al., 2018) and that siblings are at a greater risk of internalising than externalising problems, due to their reluctance to burden their parents for attention (Vermaes et al., 2012). Parents may also be more likely to identify externalising than internalising disorders in their children on the SDQ (Van der Meer et al., 2008). Research also suggests impacts on siblings’ quality of life may be greater when assessed by self-report rather than parent report (Dinleyici et al., 2020). Reliance on parent-reported measures may mean that needs of siblings may therefore have been underestimated in this study.

Almost two-thirds of attending siblings scored above clinical threshold on measures of mental health. This adds to previous literature demonstrating elevated emotional and behavioural difficulties in siblings of children with LTCs (Martinez et al., 2022; Sharpe and Rossiter, 2002; Vermaes et al., 2012) and suggests value in identifying mental health difficulties in this group. Quality of life in siblings was higher than baseline quality of life scores for children with chronic illness attending the drop-in centre (Catanzano et al., 2021). However, this may be due to children with chronic illness having lower physical functioning-related quality of life than siblings. In siblings, scores on emotional functioning (35) were comparatively lower than physical functioning (90.63), where higher scores indicate a better quality of life. A recent study of secondary school pupils in the Netherlands similarly found mean physical functioning subscale scores in 8–12 year-olds around 90, but emotional functioning was greater than 70 (Hijkoop et al., 2021). Siblings in this present study therefore appear to have poorer emotional functioning than children and young people in the general population.

Most families reported that they found the drop-in centre helpful, in line with results from children with chronic illness (Catanzano et al., 2021). As in the wider study, a central strength of this drop-in centre lies in its practicality – families are being offered assessment and treatment through flexible provision (self-referral, amount, mode of delivery and timing) that is acceptable and can fit in with family demands at an extremely stressful time. Such a flexible approach has been advocated elsewhere for families of children with chronic illness (Morawska et al., 2015). For siblings, interventions can be delivered remotely meaning that they do not need to be taken out of school, which may attenuate the adverse effects of missing school, which is common in siblings of children with LTCs (Gan et al., 2017). In future, such centres could be virtual, for example, using video conferencing software, which may improve access to assessment and intervention (Krausz et al., 2016).

Siblings received a range of levels of treatment ranging from signposting and referral to six sessions of guided self-help, demonstrating personalised intervention. Overall, there was a large effect size for mental health symptoms (d = 0.84; i.e. the average person’s score at post-intervention is 0.84 standard deviations above the average score at baseline). Effect sizes greater than 0.8 are considered large, although this does not account for clinical or practical importance of effects for different outcome measures (Eisen et al., 2007). For those with baseline and follow-up SDQ measures, mean score reduced from 18, which is in the ‘high’ range, to 15, which is in the ‘slightly raised’ range, suggesting clinically important change (Wolpert et al., 2015). These effect sizes are comparable to or larger than other brief and/or low-intensity interventions in children and young people (Bennett et al., 2019), including children with chronic illness (Catanzano et al., 2020, 2021). However, it is not possible to conclude that this was due to the intervention due to small sample size and lack of control group.

## Study limitations

These results are encouraging but the overall study sample is small, particularly for completion of quality-of-life measures. The centre provided a variety of interventions and there was no comparison group, which makes the specificity of intervention difficult to establish. Other confounders include time since sibling diagnosis, age and developmental stage and life events. Further research is needed to identify mechanisms of change of the drop-in centre as a whole, or specific interventions offered. We do not know whether identified change was due to some siblings having a parent and/or sibling with LTCs who also had intervention for mental health difficulties. Improved mental and/or physical health of the child with LTCs may have reduced family strain (Incledon et al., 2015). There may have been an indirect effect of intervention in family on siblings’ mental health, as parent/sibling and child mental health are associated (Incledon et al., 2015; Ma et al., 2015; Van Der Bruggen et al., 2008). Importantly, the child mental health outcome is parent-reported scores on the SDQ which may underestimate internalising difficulties in siblings and is likely to be confounded by parental mental health (Sharpe and Rossiter, 2002).

Future research should use independent reporting of child and parent mental health. Future research may also benefit from objective measures and full diagnostic interviews. A separate qualitative study focused on siblings’ experiences may enhance understanding of the mechanisms of change, particularly regarding the different interventions offered by the drop-in centre. It may also be helpful to understand the level of disability of their siblings LTC as this may have an impact on siblings’ experiences.

In addition, recruitment for this study took place during the COVID-19 pandemic, when hospital systems and mental health services were significantly disrupted. Although the study offered flexible and virtual options, this may have affected the data collected.

## Implications for practice

This is one of few studies exploring identification and support for the mental health of siblings of children with LTCs (McKenzie Smith et al., 2018). This pragmatic study aimed to investigate the feasibility, acceptability and preliminary effectiveness of a mental health drop-in centre in a paediatric hospital. All siblings were not routinely screened for mental health difficulties. Whilst such an approach appears to be effective and valued by the families who use it, the small number of siblings attending the drop-in centre in this pilot suggests that it is not sufficient for identifying all needs in siblings and comprehensive screening may be needed. Clinicians could explore sibling mental health in their first assessment session, perhaps using a screening measure. Measuring sibling mental health routinely may increase recognition of mental health difficulties in this group as well as aid understanding of the proportion of siblings with mental health needs and the relationship between siblings’ and the index child with the LTC and their parent/carers’ mental health.

There are many benefits of the colocation of services, the accessibility and opportunistic nature that may capture the siblings requiring mental health input that would otherwise not have received help. While we are not able to fully attribute improvement in measures to this service, it is encouraging.

Self-referral may also increase access to support for underrepresented groups. The wider project of 186 participants found that participants recruited to the project broadly matched patients seen within the wider hospital with respect to gender, ethnicity and index of multiple deprivation decile. Additionally, participants from Asian and Black backgrounds were overrepresented amongst participants recruited relative to nation-wide data from Child and Adolescent Mental Health Services (Catanzano et al., 2021). However, our exclusion of participants without a sufficient grasp of English to facilitate engagement with assessment and treatment for the purposes of the pilot will have impacted these results and future research should seek to include these young people. There are significant barriers to treating siblings, particularly around funding and governance since siblings are not the index child which is likely to restrict hospitals offering support/referring siblings elsewhere. Governance issues and care pathways need to be considered, including siblings’ medical records having to be attached to the index patient, before services can routinely be offered to siblings. It may be beneficial to make parents aware that siblings can successfully have their mental health needs met with brief treatment.

## Conclusion

The aim of this study was to explore the feasibility, acceptability and preliminary effectiveness of a drop-in centre for supporting the mental health of siblings of children with LTCs. Overall, recruitment, measures and intervention appeared feasible and acceptable. Regarding preliminary effectiveness, siblings demonstrated a decrease in mental health symptoms and an increase in quality of life following intervention. However, relatively few siblings were seen by the drop-in centre, and future research should examine the feasibility of routine screening for mental health difficulties in siblings. Overall, this study points to the feasibility and value of assessing siblings who are experiencing emotional and behavioural difficulties and providing them with an appropriate level of intervention that is accessible, effective and acceptable to families. Co-location of a children and young people’s mental health service that can provide a drop-in service capacity on site may be particularly beneficial.
